# Do the flash-lag effect and representational momentum involve similar extrapolations?

**DOI:** 10.3389/fpsyg.2013.00290

**Published:** 2013-05-23

**Authors:** Timothy L. Hubbard

**Affiliations:** Department of Psychology, Texas Christian UniversityFort Worth, TX, USA

**Keywords:** flash-lag effect, representational momentum, displacement, spatial mislocalization, spatial cognition

## Abstract

In the flash-lag effect (FLE) and in representational momentum (RM), the represented position of a moving target is displaced in the direction of motion. Effects of numerous variables on the FLE and on RM are briefly considered. In many cases, variables appear to have the same effect on the FLE and on RM, and this is consistent with a hypothesis that displacements in the FLE and in RM result from overlapping or similar mechanisms. In other cases, variables initially appear to have different effects on the FLE and on RM, but accounts reconciling those apparent differences with a hypothesis of overlapping or similar mechanisms are suggested. Given that RM is simpler and accounts for a wider range of findings (i.e., RM involves a single stimulus rather than the relationship between two stimuli, RM accounts for displacement in absolute position of a single stimulus and for differences in relative position of two stimuli), it is suggested that (at least some cases of) the FLE might be a special case of RM in which the position of the target is assessed relative to the position of another stimulus (i.e., the flashed object) rather than relative to the actual position of the target.

If observers view a moving target and a flashed (i.e., briefly presented) stationary object is presented in alignment with that moving target, the flashed object appears to lag behind the moving target. This is referred to as the *flash-lag effect* (FLE; Nijhawan, 1994; for review, Maus et al., 2010; Hubbard, in press-a). If observers view a moving target, the remembered final position of that target is shifted in the direction of target motion. This is referred to as *representational momentum* (RM; Freyd and Finke, 1984; for review, Hubbard, 2005, 2010). In the FLE (e.g., Shi and de'Sperati, 2008) and in RM (e.g., Hubbard, 1990), the represented position of a moving target is displaced forward, and the FLE (Nijhawan, 1994, 2008) and RM (Finke et al., 1986; Hubbard, 2005) have each been suggested to reflect compensation for delays in neural processing times and adaptation for real-time interaction with environmental stimuli. Surprisingly, there has been little comparison of the FLE and RM. Apparent similarities and differences of the FLE and RM are considered here, and it is suggested that displacement of the moving target in the FLE or in RM involves overlapping or similar mechanisms, or more radically, that the FLE is a special case of RM in which the represented position of the moving target is assessed relative to another object rather than relative to the actual target position.

## Apparent similarities of the FLE and RM

There are numerous apparent similarities of the FLE and RM. The existence of such similarities is consistent with a hypothesis that the FLE and RM result from overlapping or similar mechanisms.

### Perceptual similarities

Perceptual similarities involve effects of (1) velocity, (2) visual field, (3) a reference point, (4) multiple modalities, and (5) crossmodal information.

#### Velocity

The FLE (Nijhawan, 1994; Brenner and Smeets, 2000; López-Moliner and Linares, 2006; Cantor and Schor, 2007; Wojtach et al., 2008) and RM (Hubbard and Bharucha, 1988; Hubbard, 1990) increase with increases in velocity of the moving target in the picture plane. The FLE (Lee et al., 2008) and RM (Hubbard, 1996) increase with increases in velocity of the moving target in depth. The FLE (Whitney et al., 2000) and RM (Finke et al., 1986) increase or decrease with acceleration or deceleration, respectively, of the moving target in the picture plane. The velocity effect is one of the most replicated effects in the FLE or RM literatures.

#### Visual field

Whether the moving target is in the left or right visual field does not consistently influence the FLE or RM, but if an effect of visual field occurs, the FLE (Kanai et al., 2004) and RM (Halpern and Kelly, 1993; White et al., 1993) are larger if the moving target is in the left visual field. Maus and Nijhawan (2009) presented variations of a horizontally moving FLE stimulus and reported a slightly greater effect of velocity on displacement of moving targets in the upper visual field, and Hubbard (2001) reported RM was larger for vertically moving targets in the lower than in the upper visual field.

#### Reference point

RM is larger if a target moves toward rather than away from a landmark, and Hubbard and Ruppel (1999) suggested RM combined with a landmark attraction effect: If RM and landmark attraction operate in the same direction (motion toward a landmark), they sum and displacement is larger, whereas if RM and landmark attraction operate in opposite directions (motion away from a landmark), they partially cancel and displacement is smaller. Similarly, the FLE is larger if a target moves toward rather than away from the fixated region (Mateeff and Hohnsbein, 1988; Shi and Nijhawan, 2008), and Brenner et al. (2006) suggested the FLE combined with a bias toward the fixated region: If the FLE and bias toward fixation operate in the same direction (motion toward fixation), they sum and target displacement is larger, whereas if the FLE and bias toward fixation operate in opposite directions (motion away from fixation), they partially cancel and target displacement is smaller.

#### Multiple modalities

Most research on the FLE and RM presented visual stimuli. However, auditory stimuli can produce a FLE (Alais and Burr, 2003; Arrighi et al., 2005) and RM (Johnston and Jones, 2006), and haptic stimuli can produce a FLE (Nijhawan and Kirschfeld, 2003) and RM (Brouwer et al., 2005). It is possible that separate modality-specific mechanisms for the FLE and RM exist, but it is more parsimonious to posit a single mechanism or small number of higher-level mechanisms produces displacement of the moving target in the anticipated direction across multiple modalities (e.g., in higher-level processes or by top-down modulation of lower-level processes, Hubbard, 2005, 2006).

#### Crossmodal information

Visual information can influence the auditory FLE (Alais and Burr, 2003) and auditory RM (Hubbard and Courtney, 2010), and auditory information can influence the visual FLE (Vroomen and de Gelder, 2004) and visual RM (Teramoto et al., 2010). Kinesthetic information can influence the visual FLE (Cai et al., 2000; Schlag et al., 2000). Such influences of crossmodal information on the FLE and on RM are not consistent with solely lower-level explanations of the FLE or RM.

### Cognitive similarities

Cognitive similarities involve effects of (1) attention and cueing, (2) conceptual knowledge of target identity, (3) control and movement planning, (4) attribution regarding the source of motion, (5) frame of reference, and (6) neural mechanisms.

#### Attention and cueing

The FLE (Sarich et al., 2007) and RM (Hayes and Freyd, 2002) increase if attention is divided between the moving target and a concurrent irrelevant stimulus. If the position of the flashed object or moving target is cued, valid cues result in a smaller FLE (Brenner and Smeets, 2000; Namba and Baldo, 2004; Shioiri et al., 2010; but see Khurana et al., 2000) and smaller RM (Hubbard et al., 2009) than do invalid cues. RM is influenced by verbal cues given prior to target presentation (Hubbard, 1994), but whether the FLE is influenced by verbal cues has not been reported. The FLE (Maiche et al., 2007) and RM (Hubbard and Ruppel, 1999) increase if the target moves toward another object, and this might reflect spatial distribution of attention.

#### Conceptual knowledge of target identity

The FLE (Noguchi and Kakigi, 2008; Nagai et al., 2010) and RM (Reed and Vinson, 1996; Vinson and Reed, 2002) can be increased or decreased by conceptual knowledge regarding the identity of the moving target. Such influences suggest the FLE and RM do not result solely from lower-level processes, but rather result from higher-level processes or top-down modulation of lower-level processes. Also, the FLE (Moore and Enns, 2004) and RM (Kelly and Freyd, 1987) are diminished if the moving target does not maintain a consistent identity.

#### Control and movement planning

The FLE is decreased if participants control presentation of the flashed object (López-Moliner and Linares, 2006), and decreased or increased if participants control target motion with a computer mouse (Ichikawa and Masakura, 2006, 2010) or robotic arm (Scocchia et al., 2009), respectively. RM is decreased if participants control the moving target (Jordan and Knoblich, 2004; Stork and Müsseler, 2004). If participants judge the position of a moving target after acquiring experience controlling target motion, RM is larger (Jordan and Hunsinger, 2008); this was attributed to effects of action planning, and such effects might similarly account for the larger FLE when participants controlled the target in Scocchia et al. The FLE (Nijhawan, 1994, 2008) and RM (Finke et al., 1986; Hubbard, 2005) aid in planning body movements and in interactions with environmental stimuli.

#### Attribution regarding the source of motion

The FLE is influenced by whether participants believe they control target motion (Ichikawa and Masakura, 2006, 2010), and RM is influenced by whether participants attribute target motion to contact from an external stimulus or to an internal source (Hubbard, in press-b; Hubbard et al., 2001). Thus, the FLE and RM are decreased if motion is attributed to a source other than the target (e.g., the participant in the FLE, contact from another stimulus in RM), and this might result from higher-level processes or top-down modulation of lower-level processes.

#### Frame of reference

Studies of the FLE usually involve judgment of relative position (but see Munger and Owens, 2004; Shi and de'Sperati, 2008; Becker et al., 2009), whereas studies of RM usually involve judgment of absolute position; however, regardless of whether relative or absolute position is judged, represented target position in the FLE and in RM is displaced in the direction of target motion. The FLE (Maiche et al., 2007) and RM are influenced by whether another stimulus provides a landmark (Hubbard and Ruppel, 1999) or surrounding context (Hubbard, 1993), and localization of the flashed object in the FLE (van Beers et al., 2001) and moving target in RM (Hubbard, 1990, 1997) are influenced by the direction of implied gravitational attraction^1^.

#### Neural mechanisms

The FLE (Maus et al., 2013) and RM (Senior et al., 2002) are disrupted by transcranial magnetic stimulation of area MT. Kimura et al. (2011) suggested visual mismatch negativity might be related to the FLE and to RM. RM activates prefrontal cortex and anterior cingulate cortex (Rao et al., 2004); surprisingly, imaging information on the FLE has not been reported (although see Nijhawan, 2008, for discussion of potentially relevant neural mechanisms). The retina appears to compute a “crude extrapolation of the object's trajectory” (Gollisch and Meister, 2010, p. 155), and this is consistent with the FLE and with RM.

## Apparent differences of the FLE and RM

There are fewer apparent differences than apparent similarities regarding the FLE and RM. In many cases, differences that initially appear inconsistent with a hypothesis of overlapping or similar mechanisms in the FLE and RM can be reconciled with that hypothesis.

### Perceptual differences

Perceptual differences involve effects of (1) oculomotor behavior, (2) environment-centered direction, (3) object-centered direction, and (4) location within the target trajectory.

#### Oculomotor behavior

The FLE (Nijhawan, 2001) and RM (Kerzel, 2000) for a continuously moving target are decreased and increased, respectively, if participants use smooth pursuit eye movements to track that target. However, such a difference does not rule out overlapping or similar higher-level mechanisms for the FLE and RM any more than differences in oculomotor behavior with continuous motion, implied motion, or frozen-action photographs rule out overlapping or similar higher-level mechanisms for RM (for discussion, Hubbard, 2005, 2006, 2010). Oculomotor behavior modulates target displacement for only some types of visual stimuli^2^, and so cannot be the sole cause of the FLE and RM with visual stimuli. Consistent with this, the FLE and RM occur with auditory, haptic, and crossmodal stimuli, and the FLE and RM are influenced by higher-level processes (Hubbard, 2005, in press-a).

#### Environment-centered direction

The FLE is not influenced by whether targets descend or ascend (Ichikawa and Masakura, 2006, 2010), but RM is larger when targets descend than when targets ascend (Hubbard, 1990, 1997). Such findings would be consistent with a hypothesis of overlapping or similar mechanisms if the absolute positions of the moving target and the flashed object in the FLE were displaced forward equal distances (preserving their relative positions, cf. Hubbard, 2008), and these displacements were larger for ascending than for descending motion; however, measures of relative position typically used to study the FLE are not sensitive to displacement in absolute position.

#### Object-centered direction

The FLE (Nagai et al., 2010) and RM (Nagai and Yagi, 2001) are smaller and larger, respectively, if a target moves forward (its typical direction of motion) rather than backward (opposite its typical direction of motion). Such findings would be consistent with a hypothesis of overlapping or similar mechanisms if the (1) flashed object and moving target were displaced in the direction of motion, (2) displacement of the flashed object and of the moving target were smaller for backward than for forward motion, and (3) decrease in displacement with backward motion was larger for the flashed object than for the moving target. The difference between the moving target and the flashed object would appear larger for backward than for forward motion, and the FLE (difference in relative positions) would look larger even though absolute displacement was smaller.

#### Location within the target trajectory

A FLE usually occurs if the flashed object is presented at the beginning or midpoint, but not at the end, of the target trajectory (Hubbard, in press-a); however, RM is measured at the end of the target trajectory (Hubbard, 2005)^3^. One possibility consistent with a hypothesis of overlapping or similar mechanisms is that at the end of the trajectory, simultaneous decay of displacement of the moving target and displacement of the flashed object preserves their relative positions, resulting in no FLE, whereas at the beginning or midpoint of the trajectory, decay of displacement of the flashed object, coupled with continuing RM for the still-moving target, results in a FLE. Alternatively, a flashed object near the end of the target trajectory might eliminate RM (Müsseler et al., 2002) or exhibit displacement similar to that of the target (Hubbard, 2008), and thus preserve the relative positions of the flashed object and moving target, resulting in no FLE.

### Cognitive differences

Cognitive differences involve effects of (1) level of processing, (2) predictability, and (3) expertise.

#### Level of processing

In the FLE, the moving target and flashed object are perceptually available during judgment, and the FLE is usually considered a lower-level perceptual phenomenon. In RM, the moving target vanishes before judgment, and RM is usually considered a higher-level cognitive phenomenon. However, higher-level cognitive variables influence the FLE (Noguchi and Kakigi, 2008; Nagai et al., 2010), and lower-level perceptual variables influence RM (Kerzel et al., 2001; Kerzel, 2002a); thus, the FLE (Hubbard, in press-a) and RM (Hubbard, 2005) each involve lower-level perceptual processes and higher-level cognitive processes. In the FLE and in RM, represented target position at the sampled time (when the flashed object appeared or moving target vanished, respectively) is displaced forward, and this might involve overlapping or similar mechanisms at a lower or higher level.

#### Predictability

Forward displacement of a moving target in the FLE (Munger and Owens, 2004; Shi and de'Sperati, 2008) and RM (Finke and Freyd, 1985; Hubbard, 1990) suggests the FLE (Nijhawan, 1994, 2008) and RM (Hubbard, 1995, 2005) reflect predictions regarding subsequent target position^4^. Similarities in effects of attention and cueing in the FLE and in RM noted earlier suggest effects of predictability should be similar in the FLE and in RM. The FLE increases if predictability of the flashed object decreases (Baldo and Namba, 2002; Vreven and Verghese, 2005). However, RM decreases or increases if predictability (certainty) regarding target position is decreased by blocking target direction (Kerzel, 2002b) or increasing target blurriness (Fu et al., 2001), respectively. Manipulation of predictability in the FLE usually involves the flashed object, whereas manipulation of predictability in RM involves the moving target; it is not clear how predictability of a flashed object would influence localization of a moving target.

#### Expertise

RM is increased for stimuli in a domain of expertise (Blättler et al., 2010, 2011). The FLE in a given domain might be compensated for by experts in that domain (Catteeuw et al., 2009), and this suggests the FLE is decreased for stimuli in a domain of expertise. Compensation for the FLE could involve smaller displacement of the moving target or larger displacement of the flashed object. Only in the former case would effects of expertise differ for FLE and RM; the latter case is consistent with effects of RM on a nearby stationary object (Hubbard, 2008) and effects of expertise on RM for a moving target.

## Conclusions

The FLE and RM involve forward displacement of the represented position of a moving target. A large group of variables have similar influences on the FLE and on RM, and this suggests the FLE and RM might arise from overlapping or similar mechanisms. A smaller group of variables appear to have dissimilar influences on the FLE and on RM, and potential ways to reconcile those differences with a hypothesis of overlapping or similar mechanisms were suggested. Interestingly, if perceived target position is assessed relative to the position of another stimulus, then displacement is usually referred to as a “flash-lag effect,” whereas if perceived target position is assessed relative to the actual target position, then displacement is usually referred to as “representational momentum.” This suggests the FLE is a special case of RM in which displacement of the moving target is assessed relative to the position of a flashed object rather than relative to the actual target position. A hypothesis that the FLE arises from overlapping or similar mechanisms as RM, or is a special case of RM, provides important constraints for theories of the FLE and of RM. Such a hypothesis also has heuristic value, as other variables that influence the FLE or RM (e.g., contrast, presence of feedback) could be predicted to have similar effects on RM and the FLE, respectively.

In defense of a mental extrapolation theory of the FLE, Nijhawan et al. (2004, p. 278) stated “a newer interpretation of a given phenomenon can be accepted over and above an existing one only if the newer interpretation is conceptually simpler (requires fewer assumptions) and/or is capable of explaining a wider class of empirical findings.” By these criteria, an explanation of (at least some examples of) the FLE as a special case of RM should be preferred: RM is simpler than the FLE (e.g., RM involves one stimulus rather than the relationship between two stimuli) and accounts for a wider range of findings (e.g., involving a single stimulus as well as involving two stimuli). Indeed, the term “representational momentum” has a longer history in referring to extrapolation of a moving target, and so it might be appropriate and more parsimonious to consider this term and mechanism when referring to automatic extrapolation of target position, regardless of whether that extrapolation is measured relative to the position of another object or relative to the actual target position. Such an approach is consistent with models of spatial representation that address the FLE and RM (e.g., Müsseler et al., 2002; Jancke and Erlhagen, 2010) and suggests the possibility of overlapping or similar mechanisms of extrapolation.

### Conflict of interest statement

The author declares that the research was conducted in the absence of any commercial or financial relationships that could be construed as a potential conflict of interest.

## Footnotes

^1^An influence of direction of implied gravitational attraction on represented position is referred to as *representational gravity* (RG; Hubbard, 1995, 1997). van Beers et al. (2001) reported flashed objects located above or below the trajectory of a horizontally moving target were displaced away from the trajectory, and this displacement was larger if flashed objects were below the trajectory. Although RG was not considered by van Beers et al., the data they reported are consistent with a combination of RG and a bias away from the trajectory: If RG and bias away from the trajectory operate in the same direction (flashed object below the trajectory), they sum and displacement is larger, whereas if RG and bias away from the trajectory operate in opposite directions (flashed object above the trajectory), they partially cancel and displacement is smaller. Similarly, Hubbard (1990, 1997) reported that horizontally moving targets were displaced downward and forward (RG and RM operate in orthogonal directions) and that forward displacement was larger for descending targets (RG and RM operate in the same direction and sum) than for ascending targets (RG and RM operate in opposite directions and partially cancel).

^2^The FLE (e.g., Rizk et al., 2009) and RM (e.g., Munger et al., 1999) also occur with implied motion (referred to as *station to station* motion in the FLE literature), and RM also occurs for frozen-action photographs (e.g., Futterweit and Beilin, 1994); neither implied motion nor frozen-action photographs evoke smooth pursuit eye movements. Oculomotor behavior involves the hardware implementation level or perhaps algorithmic level, whereas RM or the FLE might be caused by a higher-level mechanism involving the computational level (for discussion, Hubbard, 2005, 2006). Moreover, even if oculomotor behavior is correlated with extrapolation, such a linkage is not evidence that oculomotor behavior is causal in generation of extrapolation.

^3^A related forward displacement of target position at the beginning of the target trajectory is referred to as the *Fröhlich effect* (for review, Kerzel, 2010), but the relationship of the Fröhlich effect with the FLE or with RM is beyond the scope of this paper. Even so, it should be noted that just as the FLE might reflect RM in which the perceived final position of the moving target is assessed relative to an external stimulus (i.e., the flashed object) rather than relative to the actual final position of the target, a FLE in a flash-initiated display might reflect a Fröhlich effect in which the perceived initial position of the moving target is assessed relative to an external stimulus (i.e., the flashed object) rather than relative to the actual initial position of the target (for discussion, Hubbard, in press-a).

^4^It should be noted that there are at least two different senses of “predict” in the displacement literature. Munger and Minchew (2002) use “predict” to refer to an explicit and deliberate judgment regarding the subsequent potential position of the target and use “remember” to refer to judgments of the final position (see also Finke and Freyd, 1985), whereas Nijhawan (2008) uses “predict” to refer to an implicit and automatic process that extrapolates the representation. In the current paper, “predict” and “predictability” are used in the sense of an implicit and automatic extrapolation.
